# Effects of pre-operative enteral immunonutrition for esophageal cancer patients treated with neoadjuvant chemoradiotherapy: protocol for a multicenter randomized controlled trial (point trial, pre-operative immunonutrition therapy)

**DOI:** 10.1186/s12885-022-09721-y

**Published:** 2022-06-13

**Authors:** Yuqin Cao, Dingpei Han, Su Yang, Yongmei Shi, Shengguang Zhao, Qianwen Jin, Jian Li, Chengqiang Li, Yajie Zhang, Weiyu Shen, Jinxian He, Mingsong Wang, Guangyu Ji, Zhigang Li, Yi He, Qixun Chen, Weitian Wei, Chun Chen, Xian Gong, Jinyi Wang, Lijie Tan, Hao Wang, Hecheng Li

**Affiliations:** 1 Department of Thoracic Surgery, Ruijin Hospital, Shanghai Jiao Tong University School of Medicine, 197 Ruijin Er Road, Shanghai, 200025 China; 2 Department of Clinical Nutrition, Ruijn Hospital, Shanghai Jiao Tong University School of Medicine, Shanghai, 200025 China; 3 Department of Radiotherapy, Ruijn Hospital, Shanghai Jiao Tong University School of Medicine, Shanghai, 200025 China; 4 Clinical Research Center, Ruijn Hospital, Shanghai Jiao Tong University School of Medicine, Shanghai, 200025 China; 5 Department of Thoracic Surgery, the Affiliated Lihuili Hospital, Ningbo University, Ningbo, 315048 China; 6 Department of Thoracic Surgery, Shanghai Ninth People’s Hospital, Shanghai Jiao Tong University School of Medicine, Shanghai, 200011 China; 7 Department of Thoracic Surgery, Shanghai Chest Hospital, Shanghai Jiao Tong University, Shanghai, 200030 China; 8 Department of Thoracic Oncological Surgery, Cancer Hospital of University of Chinese Academy of Sciences, Zhejiang Cancer Hospital, Hangzhou, 310022 China; 9 Department of Thoracic Surgery, Fujian Medical University Union Hospital, Fuzhou, 350001 China; 10 Department of Thoracic Surgery, Shanghai East Hospital, Tongji University School of Medicine, Shanghai, 200120 China; 11 Department of Thoracic Surgery, Zhongshan Hospital, Fudan University, Shanghai, 200032 China

**Keywords:** Esophageal neoplasms, Neoadjuvant therapy, Nutrition therapy, Combined modality therapy, Minimally invasive surgical procedures, Complications, Survival, Quality of life

## Abstract

**Background:**

Neoadjuvant chemoradiation followed by esophagectomy has been established as the first-line treatment for locally advanced esophageal cancer. Postoperative enteral nutrition has been widely used to improve perioperative outcomes. However, whether to implement preoperative nutritional intervention during neoadjuvant therapy is yet to be verified by prospective studies.

**Methods:**

POINT trial is a multicenter, open-labeled, randomized controlled trial. A total of 244 patients with surgically resectable esophageal cancer are randomly assigned to nutritional therapy group (arm A) or control group (arm B) with a 2:1 ratio. Both groups receive neoadjuvant chemotherapy with concurrent radiotherapy based on the CROSS regimen followed by minimally invasive esophagectomy. The primary endpoint is the rate of nutrition and immune-related complications after surgery. Secondary endpoints include completion rate of neoadjuvant chemoradiation and related adverse events, rate of pathological complete response, perioperative outcomes, nutritional status, overall survival, progression-free survival and quality of life.

**Discussion:**

This trial aims to verify whether immunonutrition during neoadjuvant chemoradiation can reduce the rate of complications and improve perioperative outcomes. Frequent communication and monitoring are essential for a multicenter investigator-initiated trial.

**Trial registration:** ClinicalTrials.gov: NCT04513418. The trial was prospectively registered on 14 August 2020, https://www.clinicaltrials.gov/ct2/show/NCT04513418.

**Supplementary information:**

The online version contains supplementary material available at 10.1186/s12885-022-09721-y.

## Background

### Rationale

Esophageal cancer ranks the sixth in mortality and the seventh in incidence among the malignancies worldwide in 2020 according to the latest report of cancer epidemiology [1]. Although the development of neoadjuvant therapy and radical esophagectomy have improved the treatment of locally advanced esophageal cancer, dysphagia and digestive tract reconstruction can cause malnutrition and infection-related complications, thus resulting in worse prognosis and quality of life [2–5].

Postoperative nutrition have proved to be effective in improving outcomes after esophagectomy [6, 7]. However, whether to provide a preoperative nutritional support for patients with surgically resectable esophageal cancer remains controversial. According to the current guidelines [8–10], preoperative nutrition is only recommended for malnourished cancer patients or those with severe nutritional risk. Clear evidence for the effects of preoperative nutrition on esophageal cancer patients, regardless of their nutritional status, is still lacking.

To answer this question, a systematic review and meta-analysis was conducted by our group [11]. The pooled analysis of 15 retrospective and prospective studies indicated that preoperative nutrition could reduce infectious complications and length of hospital stay after surgery. The immunonutrition, defined as specific formula enriched with immunomodulating substrates such as arginine, ω-3 fatty acids, and ribonucleotides [8], held more advantages over standard nutrition in the subgroup analyses. However, only 9 randomized controlled trials (RCTs) were found to focus on the preoperative nutrition of esophageal cancer, all of which had a short nutritional intervention lasting for only 3 days to 2 weeks [12–20]. Therefore, well-designed prospective studies are in demand in the new era of neoadjuvant therapy for esophageal cancer.

### Objectives

This prospective RCT will evaluate the effects of preoperative enteral immunonutrition on esophageal cancer patients undergoing neoadjuvant chemoradiation. The purpose of this study is to determine whether immunonutrition supply during neoadjuvant therapy can improve the rate of complications and other perioperative outcomes.

## Methods

### Trial design

**Fig. 1:**
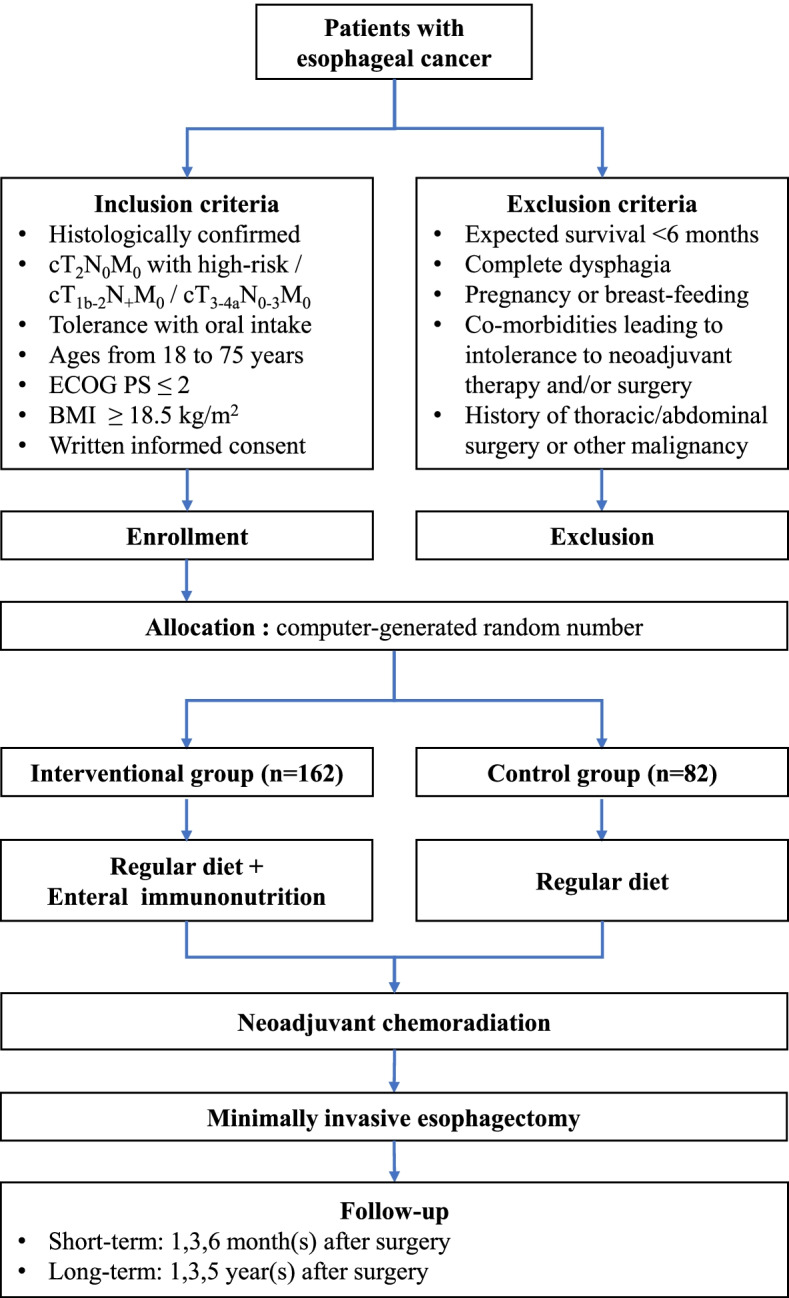
Flowchart of trial design

This is a prospective, open-labeled trial in which every eligible patient is randomly assigned to nutritional therapy group (arm A) or control group (arm B) with a 2:1 allocation ratio. Figure 1 shows the design of the POINT (Pre-Operative ImmunoNutrition Therapy) trial.

### Participants

To investigate the effects of enteral immunonutrition therapy during neoadjuvant chemoradiation on postoperative outcomes, a total of 244 patients with resectable esophageal cancer will be recruited from 8 high-volume tertiary hospitals in China. Written informed consent is obtained from each potential participant by the doctors in the participating centers.

### Inclusion criteria

1. Histologically confirmed esophageal cancer
2. Staging as cT_2_N_0_M_0_ with high-risk lesions (lymphovascular invasion, ≥ 3 cm, poorly differentiated) / cT_1b-2_ N + M_0_ / cT_3-4a_N_0-3_M_0_ with the need of neoadjuvant therapy before radical esophagectomy
3. Tolerance with oral intake (at least fluid diet)
4. Ages from 18 to 75 years
5. Eastern Cooperative Oncology Group (ECOG) performance status (PS) ≤ 2
6. Body Mass Index (BMI) ≥ 18.5 kg/m^2^ before recruitment
7. Patient’s approval and written informed consent

### Exclusion criteria

1. Expected survival time less than 6 months
2. Complete dysphagia
3. Pregnant or breast-feeding women
4. Unable to obey the interventions because of any reasons
5. Serious co-morbidities (cardiac, pulmonary, liver, kidney, brain, hematologic, endocrine and other diseases) in patients who cannot tolerate neoadjuvant therapy and/or surgery
6. History of previous thoracic or abdominal surgery
7. History of other malignant tumor (previous or current)

## Interventions

### Nutritional therapy group (arm A)

Patients in the interventional group receive 600 ml (200 ml per bottle, three times a day) immune-enhanced enteral nutritional emulsion Rui Neng® (Fresenius Kabi SSPC, China) enriched with ω-3 fatty acids and ribonucleotides per day during the neoadjuvant chemoradiation, given by oral intake, nasogastric feeding tube or jejunostomy. Meanwhile, oral intake is encouraged to reach 25–30 kcal/kg through regular diet. Nutrition assessment and consultation is provided to improve adherence to the interventions. Allergy or intolerance to the nutritional emulsion leading to < 60% of the programmed dose lasting for ≥ 7 days will be regarded as drop out.

### Control group (arm B)

Patients in the control group are encouraged to intake 25–30 kcal/kg through regular diet without supplemental nutritional support before esophagectomy. Nutrition assessment and consultation is provided to optimize the diet structure of the patients.

### Neoadjuvant chemoradiation followed by minimally invasive esophagectomy (both arms)

Both groups receive neoadjuvant chemotherapy with concurrent radiotherapy based on the CROSS regimen [21]. Chemotherapy is administered weekly for 5 weeks, with a combination of carboplatin (area under the curve [AUC] = 2) and paclitaxel or nab-paclitaxel (50 mg/m^2^). Radiotherapy with a total dose of 41.4 Gy is delivered in 23 fractions, 5 days per week.

After 4–6 weeks of neoadjuvant chemoradiation, participants will be reevaluated for any contraindications and receive minimally invasive esophagectomy (MIE) if appropriate. MIE is defined as thoracoscopic and/or laparoscopic two-incision (Ivor-Lewis) or three-incision (McKeown) esophagectomy with radical lymphadenectomy. A minimum of 15 resected LNs are recommended. The detailed approach is chosen by surgeons based on the patients’ conditions and the centers’ customs.

## Outcomes

The primary outcome is the rate of nutrition and immune-related complications up to 30 days after surgery. These include gastrointestinal complications (anastomotic leakage, gastrointestinal dysfunction), metabolic complications (electrolyte disturbances, liver or renal dysfunction) and infectious complications (wound infection, catheter-related infection, pneumonia, sepsis, or other infections requiring antibiotics).

The secondary endpoints include the following outcomes:

1. Neoadjuvant therapy measures: completion rate of neoadjuvant chemoradiation, adverse events during neoadjuvant chemoradiation, rate of pathological complete response (pCR).
2. Peri-operative measures: blood loss, operation time, rate of surgery-related complications (conversion to open surgery, recurrent nerve injury, cardiac and cerebrovascular accident), length of hospital stay, hospitalization costs.
3. Nutrition and immune-related changes measured from the beginning of neoadjuvant chemoradiation to 6 months after surgery: weight loss, Scored Patient-Generated Subjective Global Assessment (PG-SGA score), white blood cells, hemoglobin, albumin, C-reactive protein, TNF-α, interleukins, IgA, IgG, IgM, fasting blood-glucose.
4. Survival and recurrence: 30-day and 90-day mortality, overall survival (OS) (time from the date of randomization to the day of death or last follow-up), progression-free survival (PFS) (time from the date of randomization to the day of tumor progression, tumor recurrence, death or last follow-up).
5. Quality of life (QoL): European Organization for Research and Treatment of Cancer (EORTC) Quality of Life Questionnaire Core-30 (QLQ-C30) and Oesophageal-18 (QLQ-OES18) scored at randomization, before surgery, 1, 3, 6 month(s) and 1, 3, 5 year(s) after surgery.

### Power and sample size calculation

The power and sample size calculation was based on the hypothesis that pre-operative immunonutrition during the neoadjuvant therapy can reduce postoperative nutrition and immune-related complications after esophagectomy. According to the previously published articles [22] and our own experience, the related complication rates were estimated at 50% in the control group and 30% in the interventional group. The required sample size of interventional and control arm (ratio = 2:1) was therefore calculated as 137 cases and 69 cases to detect the reduction in related complications from 50 to 30% based on a bilateral significance level (*α*) of 0.05 and a power of test (1-*β*) of 0.80. Considering an estimated drop rate of 15%, the minimum sample size of this study is 244 patients, 162 cases in the interventional group and 82 in the control group.

All the esophageal cancer patients in the outpatient and inpatient service are screened for eligibility. Recruiting announcements are also published on the website of the participating center to promote recruitment.

### Allocation

After signing the informed consent, every eligible participant is randomized into nutritional therapy group or control group (ratio 2:1) based on a computer-generated random number stratified by institution. Sequentially numbered, opaque, sealed envelopes are distributed to all the study sites by the sponsor to conceal the sequence until interventions are assigned. The allocation sequence is generated by a statistician (JL). The enrollment and assignment of participants are implemented by each study site.

### Blinding

This is an open-labeled study, so trial participants and care providers are not blinded. However, outcome assessors will be masked with assignment to interventions.

### Assessment and follow-up

The time schedule of enrolment, interventions, assessments and follow-ups is summarized in Additional file 1.

#### Pre-treatment assessment

For the sake of eligibility screen, medical history, physical examination, demographic information, upper gastrointestinal endoscopy with tissue biopsy, thoracic and abdominal computed tomography (CT), and ultrasonography of abdominal organs as well as superficial lymph nodes (LNs) are routinely assessed before any treatment. After enrolment, nutritional assessments (including PG-SGA score, body weight and oral intake recording) are performed prior to neoadjuvant chemoradiation along with QoL evaluation (EORTC QLQ-C30 and QLQ-OES18 questionnaires) and laboratory tests (blood routine, biochemistry, immunoglobulin, cytokine and tumor marker).

#### Pre-operative evaluation

After 4–6 weeks of neoadjuvant chemoradiation, participants are reevaluated for the clinical response and any contraindications to esophagectomy. Physical examination, thoracic and abdominal CT scan, ultrasonography of abdomen and superficial LNs, nutritional assessments, laboratory tests and QoL evaluations are recorded.

#### Post-operative follow-up

The first 3 follow-ups are performed 1 month, 3 months and 6 months after surgery, including physical examination, nutritional assessments, laboratory tests, CT scan and QoL evaluations. Long-term follow-ups are performed 1 year, 3 years and 5 years after surgery, including physical examination, nutritional and QoL assessments as well as CT scan and other examinations for detecting recurrence. Telephone interview will be supplied to promote completion of follow-up.

#### Adverse events

All the adverse events (AE) and complications will be recorded through the study period, whether caused by neoadjuvant chemoradiation, nutritional intervention or surgery. Serious adverse events (SAE) should be reported to the principal investigator and sponsor within 24 h. Clinical trial insurance is purchased for every participant to compensate those who suffer harm from trial participation.

#### Translational research

Peripheral blood and tissue samples are collected for future translational research. Blood samples are collected in two 5 ml EDTA tubes before the start of neoadjuvant treatment and at the time of pre-operative evaluation, respectively. Tissue samples are collected before treatment (if available) and during the radical surgery, respectively.

### Data collection and monitoring

All data collected by the participating sites will be recorded in an electronic data capture (EDC) system (https://h6world.cn). To protect confidentiality, only authorized and trained investigators will have access to the data of enrolled patients. Any protocol amendments will be reviewed by the ethical committee and communicated to the participating centers after approval. Auditing will be performed every 6 months until the end of the trial.

### Statistical methods

Data will be analyzed according to the ‘intention to treat’ principle. The statistical analyses will be conducted by using R software (version 4.1.2, R Foundation for Statistical Computing, Vienna, Austria). For the primary endpoint, Pearson’s Chi-squared test or Fisher’s exact test will be used to compare the complication rate between the two groups. Regarding secondary outcomes, categorical variables will be presented as number (percentage) and analyzed as the primary endpoint. Normally distributed continuous variables will be presented as mean ± standard deviation (SD) and compared using Student’s *t*-test. In case of non-normal distribution, continuous variables will be presented as median (interquartile range [IQR]) and compared using Wilcoxon rank-sum test. Time-to-event data will be estimated by Kaplan–Meier method and analyzed by log-rank test as well as Cox proportional hazard model.

Subgroup analyses will be performed by stratifying sex, age, BMI, region, ECOG PS, tumor stage, histology and surgical procedure. *P* < 0.05 is considered to be statistically significant. Interim analysis will be conducted when 50% of the estimated patients are recruited. Missing data will be dealt with pairwise deletion.

### Current status

This study was approved by the Ethics Committee of Shanghai Jiao Tong University School of Medicine Affiliated Ruijin Hospital (RJ 2019–198) on 24 September 2019 and registered on ClinicalTrials.gov (NCT04513418) before recruitment. The current protocol version is Version 2.0 (November 2020). The recruitment was started on 10 November 2020. The POINT trial is still at the stage of recruiting as 36 patients have been recruited until 14 March 2022. The results of primary and secondary outcomes will be published after the recruitment and follow-up.

## Discussion

Esophageal cancer is a type of malignant tumor with high disease burden, especially in East Asia [23]. The treatment of esophageal cancer has greatly developed in the past several decades. For early esophageal cancer, endoscopic resection and/or esophagectomy have been widely discussed [24–28]. For locally advanced esophageal cancer, the CROSS trial [21] and NEOCRTEC5010 trial [29] have established neoadjuvant chemoradiation followed by esophagectomy as the first-line therapy. For metastatic esophageal cancer, new evidence has come up to promote early interdisciplinary care including nutritional and psychological interventions [30]. Shedding light on the multidisciplinary team (MDT) treatment for surgically resectable esophageal cancer, the POINT trial aims to provide convincing evidence for nutritional intervention during neoadjuvant multimodality therapy based on our previous study [11].

However, several practical and operational limitations exist when performing this multicenter RCT. Firstly, the compliance and retention of participants may be unsatisfactory due to the characteristics of nutritional intervention. The oral intake and tolerance with enteral nutritional emulsion may vary with the progression of disease. Secondly, confounding factors should never be neglected as the surgical procedures and techniques play a critical role in postoperative outcomes. Randomization and subgroup analyses may relieve the bias to a certain extent. Thirdly, multicenter participation may add to difficulties in long-term follow-up since this is an investigator-initiated trial (IIT). Therefore, frequent communications and monitoring are of great importance during the conduction of this trial. Routine follow-up and MDT care will also help improve the compliance of patients.

## Supplementary information

**Additional file1:** **Table S1**. Time schedule of enrolment, interventions, and assessments.

## Data Availability

Data sharing is not applicable to this article as no datasets were generated or analyzed during the current study.

## Abbreviations

AE: Adverse event
AUC: Area under the curve
BMI: Body mass index
CT: Computed tomography
ECOG: Eastern Cooperative Oncology Group
EDC: Electronic data capture
EORTC: European Organization for Research and Treatment of Cancer
IIT: Investigator-initiated trial
IQR: Interquartile range
LN: Lymph node
MIE: Minimally invasive esophagectomy
OS: Overall survival
pCR: Pathological complete response
PFS: Progression-free survival
PG-SGA score: Scored Patient-Generated Subjective Global Assessment
POINT: Pre-operative immunonutrition therapy
PS: Performance status
QoL: Quality of life
QLQ-C30: Quality of Life Questionnaire Core-30
RCT: Randomized controlled trial
SAE: Serious adverse event
SD: Standard deviation

## References

[1] Sung H, Ferlay J, Siegel RL, Laversanne M, Soerjomataram I, Jemal A (2021). Global Cancer Statistics 2020: GLOBOCAN Estimates of Incidence and Mortality Worldwide for 36 Cancers in 185 Countries. CA Cancer J Clin.

[2] Mitzman B, Schipper PH, Edwards MA, Kim S, Ferguson MK (2018). Complications After Esophagectomy Are Associated With Extremes of Body Mass Index. Ann Thorac Surg.

[3] Heneghan HM, Zaborowski A, Fanning M, McHugh A, Doyle S, Moore J, et al. Prospective Study of Malabsorption and Malnutrition After Esophageal and Gastric Cancer Surgery. Ann Surg. 2015;262:803–7; discussion 807–808. 10.1097/SLA.0000000000001445 26583669

[4] Ouattara M, D’Journo XB, Loundou A, Trousse D, Dahan L, Doddoli C (2012). Body mass index kinetics and risk factors of malnutrition one year after radical oesophagectomy for cancer. Eur J Cardio-Thorac Surg Off J Eur Assoc Cardio-Thorac Surg.

[5] D’Journo XB, Ouattara M, Loundou A, Trousse D, Dahan L, Nathalie T (2012). Prognostic impact of weight loss in 1-year survivors after transthoracic esophagectomy for cancer. Dis Esophagus Off J Int Soc Dis Esophagus.

[6] Ligthart-Melis GC, Weijs PJM, te Boveldt ND, Buskermolen S, Earthman CP, Verheul HMW (2013). Dietician-delivered intensive nutritional support is associated with a decrease in severe postoperative complications after surgery in patients with esophageal cancer. Dis Esophagus.

[7] Berkelmans GHK, Fransen LFC, Dolmans-Zwartjes ACP, Kouwenhoven EA, van Det MJ, Nilsson M (2020). Direct Oral Feeding Following Minimally Invasive Esophagectomy (NUTRIENT II trial): An International, Multicenter. Open-label Randomized Controlled Trial Ann Surg.

[8] Weimann A, Braga M, Carli F, Higashiguchi T, Hübner M, Klek S (2021). ESPEN practical guideline: Clinical nutrition in surgery. Clin Nutr Edinb Scotl.

[9] Ajani JA, D’Amico TA, Bentrem DJ, Chao J, Corvera C, Das P, et al. Esophageal and Esophagogastric Junction Cancers, Version 2.2019, NCCN Clinical Practice Guidelines in Oncology. J Natl Compr Cancer Netw JNCCN. 2019;17:855–83. 10.6004/jnccn.2019.0033 31319389

[10] August DA, Huhmann MB, American Society for Parenteral and Enteral Nutrition (A.S.P.E.N.) Board of Directors. A.S.P.E.N. clinical guidelines: nutrition support therapy during adult anticancer treatment and in hematopoietic cell transplantation. JPEN J Parenter Enteral Nutr. 2009;33:472–500. 10.1177/0148607109341804 19713551

[11] Cao Y, Han D, Zhou X, Han Y, Zhang Y, Li H. Effects of preoperative nutrition on postoperative outcomes in esophageal cancer: a systematic review and meta-analysis. Dis Esophagus Off J Int Soc Dis Esophagus. 2022;35:doab028. 10.1093/dote/doab028 33969399

[12] Aiko S, Kumano I, Yamanaka N, Tsujimoto H, Takahata R, Maehara T (2012). Effects of an immuno-enhanced diet containing antioxidants in esophageal cancer surgery following neoadjuvant therapy. Dis Esophagus Off J Int Soc Dis Esophagus.

[13] Lu Q, Zheng K, Zhang P (2013). Effect of preoperative enteral nutrition on postoperative infections and nutritional indices in esophageal cancer patients with esophageal stenosis. World Chin J Dig.

[14] Sultan J, Griffin SM, Di Franco F, Kirby JA, Shenton BK, Seal CJ (2012). Randomized clinical trial of omega-3 fatty acid-supplemented enteral nutrition versus standard enteral nutrition in patients undergoing oesophagogastric cancer surgery. Br J Surg.

[15] Mudge LA, Watson DI, Smithers BM, Isenring EA, Smith L, Jamieson GG (2018). Multicentre factorial randomized clinical trial of perioperative immunonutrition versus standard nutrition for patients undergoing surgical resection of oesophageal cancer. Br J Surg.

[16] Kanekiyo S, Takeda S, Iida M, Nishiyama M, Kitahara M, Shindo Y (2019). Efficacy of perioperative immunonutrition in esophageal cancer patients undergoing esophagectomy. Nutr Burbank Los Angel Cty Calif.

[17] Kitagawa H, Namikawa T, Yatabe T, Munekage M, Yamasaki F, Kobayashi M (2017). Effects of a preoperative immune-modulating diet in patients with esophageal cancer: a prospective parallel group randomized study. Langenbecks Arch Surg.

[18] Healy LA, Ryan A, Doyle SL, Ní Bhuachalla ÉB, Cushen S, Segurado R (2017). Does Prolonged Enteral Feeding With Supplemental Omega-3 Fatty Acids Impact on Recovery Post-esophagectomy: Results of a Randomized Double-Blind Trial. Ann Surg.

[19] Ryan AM, Reynolds JV, Healy L, Byrne M, Moore J, Brannelly N (2009). Enteral nutrition enriched with eicosapentaenoic acid (EPA) preserves lean body mass following esophageal cancer surgery: results of a double-blinded randomized controlled trial. Ann Surg.

[20] Sakurai Y, Masui T, Yoshida I, Tonomura S, Shoji M, Nakamura Y, et al. Randomized clinical trial of the effects of perioperative use of immune-enhancing enteral formula on metabolic and immunological status in patients undergoing esophagectomy. World J Surg. 2007;31:2150–7; discussion 2158–2159. 10.1007/s00268-007-9170-8 17653789

[21] van Hagen P, Hulshof MCCM, van Lanschot JJB, Steyerberg EW, van Berge Henegouwen MI, Wijnhoven BPL (2012). Preoperative chemoradiotherapy for esophageal or junctional cancer. N Engl J Med.

[22] Low DE, Kuppusamy MK, Alderson D, Cecconello I, Chang AC, Darling G (2019). Benchmarking Complications Associated with Esophagectomy. Ann Surg.

[23] Pennathur A, Gibson MK, Jobe BA, Luketich JD (2013). Oesophageal carcinoma Lancet Lond Engl.

[24] Maruyama S, Okamura A, Imamura Y, Kanamori J, Kanie Y, Takahashi K (2021). Comparison of Outcomes Between Additional Esophagectomy After Noncurative Endoscopic Resection and Upfront Esophagectomy for T1N0 Esophageal Squamous Cell Carcinoma. Ann Surg Oncol.

[25] Liu Z, Li Z (2021). Letter to the Editor: Comparison of Outcomes Between Additional Esophagectomy After Noncurative Endoscopic Resection and Upfront Esophagectomy for T1N0 Esophageal Squamous Cell Carcinoma. Ann Surg Oncol.

[26] Maruyama S, Okamura A, Watanabe M (2021). Author’s Reply: Comparison of Outcomes Between Additional Esophagectomy After Noncurative Endoscopic Resection and Upfront Esophagectomy for T1N0 Esophageal Squamous Cell Carcinoma. Ann Surg Oncol.

[27] Liu Z, Zhang J, Su Y, Pan J, Yang Y, Huang B (2021). Additional Esophagectomy Following Noncurative Endoscopic Resection for Early Esophageal Squamous Cell Carcinoma: A Multicenter Retrospective Study. Ann Surg Oncol.

[28] Liu Z, Zhao J, Li Z (2021). ASO Author Reflections: Is Esophagectomy Necessary after Noncurative Endoscopic Resection for Early Esophageal Squamous Cell Carcinoma?. Ann Surg Oncol.

[29] Yang H, Liu H, Chen Y, Zhu C, Fang W, Yu Z (2018). Neoadjuvant Chemoradiotherapy Followed by Surgery Versus Surgery Alone for Locally Advanced Squamous Cell Carcinoma of the Esophagus (NEOCRTEC5010): A Phase III Multicenter, Randomized, Open-Label Clinical Trial. J Clin Oncol Off J Am Soc Clin Oncol.

[30] Lu Z, Fang Y, Liu C, Zhang X, Xin X, He Y (2021). Early Interdisciplinary Supportive Care in Patients With Previously Untreated Metastatic Esophagogastric Cancer: A Phase III Randomized Controlled Trial. J Clin Oncol Off J Am Soc Clin Oncol.

