# Supplementary material for: Effects of pre-operative enteral immunonutrition for esophageal cancer patients treated with neoadjuvant chemoradiotherapy: protocol for a multicenter randomized controlled trial (point trial, pre-operative immunonutrition therapy)
Source: BMC Cancer. 2022 Jun 13;22:650. doi: 10.1186/s12885-022-09721-y (PMC9190085; doi:10.1186/s12885-022-09721-y)
Supplement: Supplementary file 1 — Additional file1: Table S1. Time schedule of enrolment, interventions, and assessments. [file 12885_2022_9721_MOESM1_ESM.docx]

Table S1. Time schedule of enrolment, interventions, and assessments.

|  | **STUDY PERIOD** | | | | | |
| --- | --- | --- | --- | --- | --- | --- |
|  | **Enrolment** | **Post-allocation** | | | | |
| **TIMEPOINT** | Baseline | Start of neoadjuvant therapy | Preoperative evaluation | Perioperative hospitalization | 1/3/6 month(s) after surgery | 1/3/5 year(s) after surgery |
| ***ENROLMENT:*** | | | | | | |
| Eligibility screen | X |  |  |  |  |  |
| Informed consent | X |  |  |  |  |  |
| Demographic  information | X |  |  |  |  |  |
| Medical history | X |  |  |  |  |  |
| Physical  examination | X | X | X | X | X | X |
| Allocation | X |  |  |  |  |  |
| ***INTERVENTIONS*:** | | | | | | |
| Oral intake  ± nutritional therapy |  | X |  |  |  |  |
| Neoadjuvant  chemoradiation |  | X |  |  |  |  |
| ***NUTRITIONAL ASSESSMENTS*:** | | | | | | |
| PG-SGA Score |  | X | X |  | X | X |
| Body weight |  | X | X | X | X | X |
| Oral intake  (% of previous diet) |  | X | X |  | X | X |
| ***LABORATORY TESTS*:** | | | | | | |
| Blood routine |  | X | X |  | X |  |
| Biochemistry |  | X | X |  | X |  |
| Immunoglobulin |  | X | X |  | X |  |
| Cytokine |  | X | X |  | X |  |
| Tumor marker |  | X | X |  | X |  |
| ***IMAGING EXAMINATIONS*:** | | | | | | |
| Endoscopy +  biopsy | X |  |  |  |  |  |
| Thoracic + abdominal  CT scan | X |  | X |  | X | X |
| Ultrasonography of  abdomen and LNs | X |  | X |  |  |  |
| ***OTHER ASSESSMENTS*:** | | | | | | |
| EORTC QoL  questionnaire |  | X | X |  | X | X |
| Adverse events and complications |  | X | X | X | X | X |
| Survival and  recurrence |  | X | X | X | X | X |
| Blood sample |  | X | X |  |  |  |
| Tissue sample | X |  |  | X |  |  |

Abbreviations: PG-SGA score, Scored Patient-Generated Subjective Global Assessment; CT, Computed tomography; LN, Lymph node; EORTC, European Organization for Research and Treatment of Cancer; QoL, Quality of life.
